# Mulberrin Confers Protection against Doxorubicin-Induced Cardiotoxicity via Regulating AKT Signaling Pathways in Mice

**DOI:** 10.1155/2022/2967142

**Published:** 2022-07-07

**Authors:** Peng Ye, Wen-Lan Li, Long-Tang Bao, Wei Ke

**Affiliations:** ^1^Department of Pharmacy, Renmin Hospital of Wuhan University, Wuhan 430060, China; ^2^Department of Anesthesiology, Renmin Hospital of Wuhan University, Wuhan, Hubei 430060, China; ^3^Day-Care Unit, The Affiliated Hospital of Inner Mongolia Medical University, Hohhot 010050, China; ^4^Department of Neurology, Renmin Hospital of Wuhan University, Wuhan 430060, China

## Abstract

Doxorubicin (DOX) is an antitumor anthracycline, but its clinical use was largely limited by its cardiac toxicity. DOX-induced oxidative damage and cardiomyocyte loss have been recognized as the potential causative mechanisms of this cardiac toxicity. Growing interests are raised on mulberrin (Mul) for its wide spectrum of biological activities, including antioxidative and anti-inflammatory properties. The aim of this study was to investigate the effect of Mul on DOX-induced heart injury and to clarify the underlying mechanism. Mice were given daily 60 mg/kg of Mul via gavage for 10 days. Mice received an intraperitoneal injection of DOX to mimic the model of DOX-related acute cardiac injury at the seventh day of Mul treatment. Mul-treated mice had an attenuated cardiac injured response and improved cardiac function after DOX injection. DOX-induced oxidative damage, inflammation accumulation, and myocardial apoptosis were largely attenuated by the treatment of Mul. Activated protein kinase B (AKT) activation was essential for the protective effects of Mul against DOX-induced cardiac toxicity, and AKT inactivation abolished Mul-mediated protective effects against DOX cardiotoxicity. In conclusion, Mul treatment attenuated DOX-induced cardiac toxicity via activation of the AKT signaling pathway. Mul might be a promising therapeutic agent against DOX-induced cardiac toxicity.

## 1. Introduction

Doxorubicin (DOX) is a widely used chemotherapy drug. This drug results in cardiotoxicity, which is manifested as a progressive and irreversible cardiomyopathy [1]. The incidence of DOX-induced cardiac injury ranges from 11% to 18%, as estimated by previous studies [2, 3]. The onset of DOX-induced cardiotoxicity can be acute, occurring within 2-3 days or be chronic until several months after the end of chemotherapy [3]. Current studies pay more attention to DOX-related chronic cardiomyopathy but lose sight of the clinical importance of DOX-induced acute cardiotoxicity. Several mechanisms are involved in the process of DOX-related toxicities including mitochondrial dysfunction, oxidative stress, inhibition of autophagy, and myocardial apoptosis [4]. Currently, there are no molecules with an actual cardioprotective effect on DOX-induced acute cardiotoxicity. It is therefore important to find an approach for preventing DOX-induced cardiotoxicity in the clinic.

DOX treatment leads to the production of free oxygen radicals and antioxidant deficiency, which causes oxidative stress in the heart [1, 4]. Oxidative stress has been established as a potential causative mechanism [5, 6]. Overexpression of antioxidant enzymes can ameliorate DOX-induced cardiotoxicity in mice [7, 8]. DOX-dependent oxidative damage induced the release of cytochrome C and subsequent activation of caspase 3, causing myocardial apoptosis [9]. The attenuation of DOX-induced myocardial apoptosis could protect against DOX-induced cardiotoxicity [10]. These findings highlighted the importance of finding a promising therapeutic strategy to inhibit oxidative damage and apoptotic cell death in DOX-related cardiotoxicity.

Mulberrin (Mul) is a natural product of Ramulus mori and has potent biological abilities, including antioxidant, anti-inflammatory, antifibrotic effects [11, 12]. Cao et al. found that Mul attenuated Parkinson's disease by activating the *β*-catenin signaling pathway in mice [13]. Mul reduced spinal cord injury-induced apoptosis and inflammation and attenuated hepatic fibrosis by targeting nuclear factor E2-related factor 2 (Nrf2) in mice [11, 12]. Mul significantly reduced blood glucose and alleviated the metabolic syndrome in rodents [14]. Based on these reports, we speculated that Mul might protect against DOX-related cardiac injury in mice. Therefore, we designed this study to explore the effect of Mul treatment on DOX-derived cardiotoxicity.

## 2. Materials and Methods

### 2.1. Materials

Mul (#62949-79-5, purity ≥ 95% as detected by HPLC) was provided by MedChemExpress (Shanghai, China), and this reagent was dissolved into 0.1% dimethyl sulfoxide (DMSO). DOX and the protein kinase B (AKT) inhibitor were purchased from Sigma-Aldrich (St. Louis, USA). DMEM and fetal bovine serum (FBS) were provided by Thermo Fisher (Shanghai, China).

### 2.2. Animals

All animal experimental procedures were approved by the Institutional Animal Care and Use Committee at Renmin Hospital of Wuhan University (Wuhan, China). C57BL/6 male mice with 8-9 weeks of age (body weight: 21-24 g) were purchased from HFK Bioscience (Beijing, China) and housed at a standard temperature (24 ± 2°C) and humidity (50–60%) under a 12 h/12 h photoperiod. Mice were orally given 60 mg/kg Mul by gavage for 10 days. At the seventh day of Mul treatment, mice received an intraperitoneal injection of DOX (20 mg/kg) to mimic the model of DOX-related acute cardiac injury. The dose of Mul was selected according to a previous study [11]. Normal saline was used instead of Mul in control groups. Three days after DOX or normal saline injection, mice were anaesthetized and sacrificed to evaluate DOX-related cardiac injury.

The adeno-associated virus 9 (AAV9) vectors carrying protein kinase B- (AKT-) dominant negative mutant (dnAKT) and GFP were provided by Hanbio Biotechnology Co. (Shanghai, China). To overexpress this mutant in the heart, mice were given a single injection of AAV9-dnAKT at a dose of 1 × 10^11^ particles per mouse via tail vein [15]. All mice were divided into 6 groups: saline+Con, DOX+Con, DOX+Mul+Con, saline+dnAKT, DOX+dnAKT, and DOX+Mul+dnAKT groups (*n* = 8 for each group). Four weeks after AAV9-dnAKT infection, these mice received 20 mg/kg DOX to mimic DOX-related acute cardiac injury. Three days after DOX or normal saline injection, all mice were sacrificed to the phenotypes.

### 2.3. Invasive Hemodynamics

Invasive hemodynamic monitoring was conducted in mice anesthetized with 2% isoflurane by a 1.0 F microtip catheter (PVR 1045), which was connected to a Millar Pressure-Volume System (MPVS-400; Millar Instruments). These data were recorded and analyzed by a PVAN analysis software.

### 2.4. Cell Culture and Treatment

H9c2 cardiomyocytes were purchased from the American Type Culture Collection (ATCC) and cultured in DMEM supplemented with 10% FBS. Only H9c2 cells at 3-5 passages were used for this experiment. At approximately 75% confluence, these cells were starved with a serum-free DMEM medium for 24 hours. After that, H9c2 cardiomyocytes were incubated in DMEM medium containing PBS, DOX (1 *μ*M), DOX (1 *μ*M) plus 25 *μ*M Mul, DOX (1 *μ*M) plus 50 *μ*M Mul, and DOX (1 *μ*M) plus 100 *μ*M Mul. Cells were harvested at 0 h, 12 h, 24 h, and 48 h. Cell viability was examined using the Cell Counting Kit-8 assay (CCK-8, #HY-K0301, MedChemExpress) according to the manufacturer's instructions. To inhibit the activation of AKT, H9c2 cardiomyocytes were pretreated with a specific AKT inhibitor (1 *μ*M) for 24 h.

H9c2 cardiomyocytes were cultured in a six-well plate. After serum starvation for 24 h, H9c2 cells were electrotransfected with nuclear factor kappa-B- (NF-*κ*B-) luc (0.03 *μ*g) with a Neon® Transfection System (pulse width: 20 ms, pulse voltage: 1700 V). After that, these cells were treated with DOX and Mul (100 *μ*M) for 48 h. At the end of this experiment, cells were lysed in 100 *μ*l of a cell lysis reagent (Promega, Madison, USA). Luciferase activity was detected with a Promega Luciferase assay reagent (#E1500).

### 2.5. Western Blotting and Quantitative Real-Time PCR

Total protein was extracted from mouse hearts and cultured cells using the RIPA Lysis Buffer [10]. NE-PER™ Nuclear and Cytoplasmic Extraction Reagent (#78833, Invitrogen, Carlsbad, CA, USA) was used to separate nuclear proteins. These proteins were separated by electrophoresis and transferred to nitrocellulose membranes and incubated with several primary antibodies against Nrf2 (#ab62352, 1 : 1000, Abcam, Cambridge, MA, USA), heme oxygenase-1 (HO-1, Abcam, #ab52947, 1 : 1000), GAPDH (Abcam, #ab9485, 1 : 1000), NF-*κ*B p65 (Abcam, #ab207297, 1 : 1000), anti-NF-*κ*B p65 phospho S536 (Abcam, #ab239882, 1 : 1000), inhibitor of kappa B alpha (I*κ*B*α*, Abcam, #ab76429, 1 : 2000), phosphorylated-I*κ*B*α* (Abcam, #ab133462, 1 : 2000), glycogen synthase kinase 3*β* (GSK3*β*, Abcam, #ab32391, 1 : 1000), phosphorylated-GSK3*β* (Abcam, #ab75814, 1 : 1000), Bax (Abcam, #ab32503, 1 : 1000), Bcl-2 (Abcam, #ab182858, 1 : 1000), mammalian target of rapamycin (mTOR Abcam, #ab32028, 1 : 1000), and phosphorylated-mTOR (Abcam, #ab109268, 1 : 1000). After that, the membranes were then hybridized with HRP-conjugated secondary antibodies (Proteintech; 1 : 5000) for 2 h at room temperature. The membranes were scanned with the Quant LAS 500 system.

Total mRNA was extracted from heart tissues with a TRIzol reagent (Invitrogen, Carlsbad, USA). The isolated RNA was reversely transcribed into complementary DNA using Advantage® RT-for-PCR Kit (#639505, Takara Bio, Kusatsu, Shiga, Japan). Quantitative PCR was conducted with an iQ5 Multi-Color Real-Time PCR Detection System (Bio-Rad, CA, USA) using the SYBR Green Real-Time PCR Master Mix kit (#QPK-201, Takara, Dalian, China).

### 2.6. Biochemical Analyses

Heart tissues were homogenized in iced PBS, and homogenates were centrifuged at 4800 g for 20 minutes to isolate the supernatant. Myocardial lactate dehydrogenase (LDH) and creatine kinase- (CK-) MB were detected by using the commercial kits. The mouse LDH ELISA kit was provided by CUSABIO (#CSB-E17733m, Wuhan, China). The CK-MB assay kit (#A032-1-1) was obtained from the Nanjing Jiancheng Biological Engineering Research Institute.

The levels of glutathione (GSH), malondialdehyde (MDA), 3-nitrotyrosine (3-NT), protein carbonyl, total SOD activity, tumor necrosis factor-*α* (TNF-*α*) level, and interleukin- (IL-) 6 level were determined using commercial kits based on the manufacturer's instructions. GSH assay kit (#A061), MDA assay kit (A003-1-2), and the total SOD activity (A001-3-2) were provided by the Nanjing Jiancheng Institute of Biotechnology (Nanjing, China). 3-NT competitive ELISA (#ab113848) was obtained from Abcam. The protein carbonyl ELISA kit was provided by Abnova (#KA6397, Taipei, Taiwan). The TNF-*α* Mouse ELISA kit (#BMS607-3TEN) was provided by Invitrogen, and the IL-6 (mouse) ELISA kit was provided by Biovision (#K4795). All assays were performed in triplicate.

### 2.7. Reactive Oxygen Species Production Measurements and Immunohistochemistry

The reactive oxygen species (ROS) was measured with 2′,7′-dichlorofluorescein-diacetate (DCFH-DA, Beyotime Institute) staining. H9c2 cells were incubated with DCFH-DA (10 *μ*M) for 60 min in 37°C, and immunofluorescence was detected via a fluorescence microplate reader and an Olympus IX53 fluorescence microscope. To detect myocardial lipid peroxidation products, 4-hydroxynonenal (4-HNE) immunohistochemistry was performed. Prepared heart sections were incubated with an anti-4-HNE antibody (1 : 200, #ab48506, Abcam) at 4°C overnight, then with secondary antibodies at 37°C for 1 h, and detected with 3,3′-diaminobenzidine, and sections were counterstained with hematoxylin. Immunohistochemistry images were captured via Aperio VERSA 8 (Leica Biosystems). All images were analyzed by a person blinded to the treatment by using Image-Pro Plus 6.0.

### 2.8. Apoptotic Detection

Frozen heart tissues were cut into sections and fixed in 4% neutral paraformaldehyde. TUNEL assay was conducted with the in situ apoptosis detection kit (Takara Bio Inc., Shiga, Japan), and these slides were observed under a fluorescence microscope. This evaluation was performed by one person who was blinded to the treatment group. Heart tissues were homogenized in iced PBS. Cardiac caspase 3 levels were detected using a Caspase-3 Colorimetric Assay Kit (Clontech, USA). DNA fragmentation assay was detected using the DNA Fragmentation Detection Kit (#AS20-4459, Agrisera) according to the manufacturer's instructions.

### 2.9. Statistical Analysis

Data are represented as mean ± standard error of mean (SEM). Statistical analysis between two groups was determined via unpaired Student's *t*-test. We used one-way analysis of variance followed by Tukey's test to compare differences between multiple groups. Differences were considered as statistically significant at *P* < 0.05.

## 3. Result

### 3.1. Mul Treatment-Attenuated DOX-Induced Oxidative Stress In Vitro

**Figure 1:**
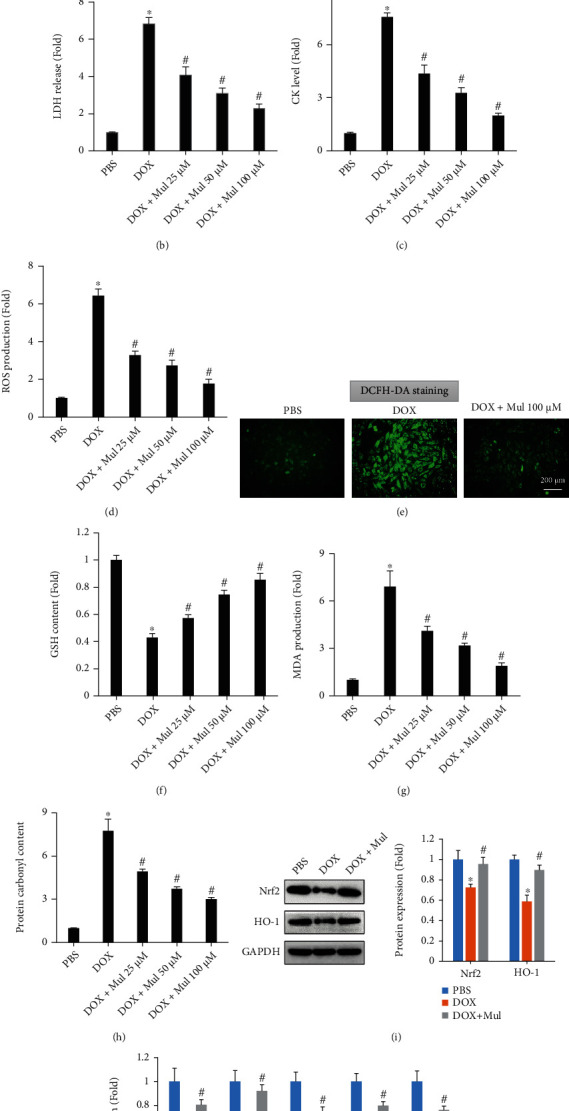
Mulberrin-suppressed DOX-induced ROS generation and oxidative stress in cardiomyocytes. Cell viability (a) and the release of LDH (b) and CK (c) were performed to estimate the toxic effects on cells in response to DOX. The ROS level (d, e), GSH content (f), MDA content (g), and protein carbonyl content (h) in the DOX-treated cells were detect to reflect oxidative damage caused by DOX. Nrf2 and HO-1 protein (i) were detected in the indicated groups. The mRNA levels of Nrf2 downstream gene products (j) were also quantified. Data are presented as mean ± SEM. *n* = 5 − 6 for each group at each time point. ^∗^*P* < 0.05 compared with the PBS group; ^#^*P* < 0.05 compared with DOX alone.

We first used a CCK-8 assay to determine the viability of DOX-treated cells. Compared with the PBS group, DOX time-dependently decreased the viability of cardiomyocytes. However, this pathological reduction was markedly attenuated by Mul treatment at either 50 or 100 *μ*M (Figure 1(a)). However, Mul at the dose of 25 *μ*M did not improve the viability of DOX-treated cells. The levels of LDH and CK in DOX-treated cells were higher than with those in the PBS-treated group. However, Mul treatment significantly inhibited the release of LDH and CK in response to DOX administration (Figures 1(b) and 1(c)). It also showed that the ROS level in DOX-treated H9c2 cells was increased. And Mul treatment dose-dependently decreased the cellular ROS level in DOX-treated cells (Figure 1(d)). DCFH-DA immunofluorescence also revealed that Mul significantly decreased DOX-induced ROS production in vitro (Figure 1(e)). In addition, Mul treatment also dose-dependently increased GSH content and decreased MDA and protein carbonyl levels in DOX-exposed cells (Figures 1(f)–1(h)). The protein expression of Nrf2 and HO-1 was significantly lower in cardiomyocytes exposed to DOX than in those exposed to PBS. The expression of Nrf2 and HO-1 exposure to DOX was higher in cardiomyocytes treated with Mul than in those treated with DOX only (Figure 1(i)). We also determined the mRNA levels of Nrf2-regulated downstream targets. The decreased mRNA levels of HO-1, SOD1, SOD2, NQO1, and CAT were obviously restored after Mul treatment (Figure 1(j)).

### 3.2. Mul Treatment-Suppressed Inflammatory Response in DOX-Incubated Cells

**Figure 2:**
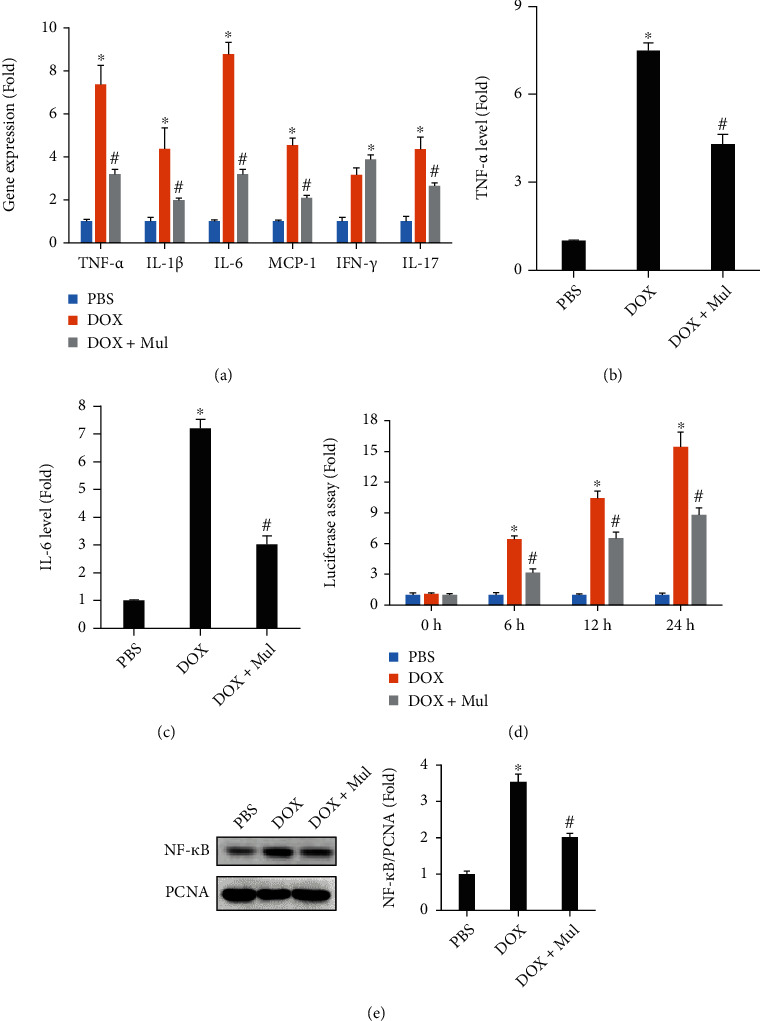
Mulberrin at the dose of 100 *μ*M attenuated the upregulation of inflammatory cytokines induced by DOX in vitro. The mRNA levels of inflammatory factors (a) were examined by qRT-PCR. The protein expression of TNF-*α* (b) and IL-6 (c) were detected by ELISA. Luciferase assay (d) was used to reflect NF-*κ*B transactivation in vitro. The nuclear accumulation of NF-*κ*B protein (e) was detected in the indicated groups. Data are presented as mean ± SEM. *n* = 5 for each group. ^∗^*P* < 0.05 compared with the PBS group; ^#^*P* < 0.05 compared with DOX alone.

The mRNA levels of several inflammatory factors, including TNF-*α*, IL-1*β*, IL-6, monocyte chemoattractant protein-1 (MCP-1), interferon *γ* (IFN-*γ*), and IL-17, in cardiomyocytes were greater in the DOX group than in the PBS group. Mul treatment (100 *μ*M) attenuated all these pathological elevations except IFN-*γ* (Figure 2(a)). Further detection the protein expression of TNF-*α* and IL-6 also reveal that Mul at the dose of 100 *μ*M suppressed the inflammatory response induced by DOX (Figures 2(b) and 2(c)). The luciferase assay also revealed that DOX induced NF-*κ*B transactivation in a time-dependent manner. And Mul at the dose of 100 *μ*M largely suppressed this activation of NF-*κ*B after DOX incubation in a time-dependent manner (Figure 2(d)). Detection of nuclear NF-*κ*B found that Mul at the dose of 100 *μ*M suppressed nuclear NF-*κ*B accumulation in DOX-treated cardiomyocytes (Figure 2(e)).

### 3.3. Mul Treatment-Attenuated Acute Cardiac Injury in DOX-Injected Mice

**Figure 3:**
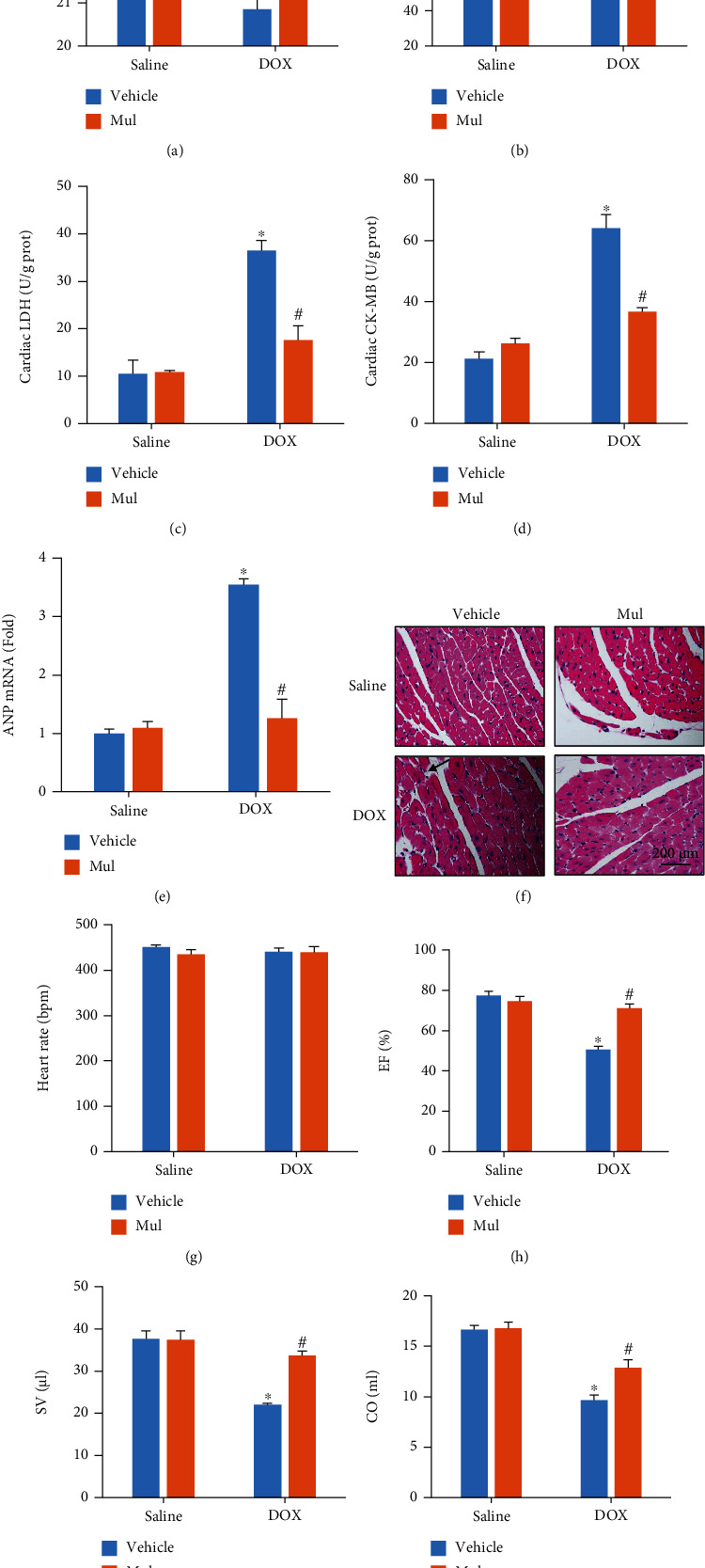
Mulberrin treatment prevented the DOX-induced cardiac dysfunction in mice. Body weight (a), heart weight (b), and myocardial LDH and CK (c, d) content were detected to evaluate DOX-induced cardiac injury. The mRNA level of ANP (e) was examined by qRT-PCR. H&E staining (f). Black line indicates myofibrillar disarrangement. Cardiac function including heart rate (g), ejection fraction (h), stroke volume (i), and cardiac output (j) was detected using an invasive hemodynamic monitoring. Data are presented as mean ± SEM. For (a, b), *n* = 10 each group; for (c–j), *n* = 6 each group. ^∗^*P* < 0.05 compared with the saline group; ^#^*P* < 0.05 compared with DOX alone.

As shown in Figures 3(a) and 3(b), body weight and heart weight declined in response to DOX injection. And these declines in DOX-treated mice were largely blocked by the treatment of Mul (Figures 3(a) and 3(b)). The release of LDH and CK-MB can reflect the severity of cardiac injury. DOX significantly upregulated LDH and CK-MB levels in heart tissues, which were alleviated by Mul treatment (Figures 3(c) and 3(d)). The detection of the mRNA level of ANP also revealed that Mul treatment significantly decreased the elevated ANP mRNA level in DOX-treated mice (Figure 3(e)). Histological detection revealed that myofibrillar disarrangement in DOX-injected mice was attenuated by Mul treatment (Figure 3(f)). Unexpectedly, DOX injection did not decrease the heart rate in mice (Figure 3(g)). Compared to those in the saline group, ejection fraction (EF), stroke volume (SV), and cardiac output (CO) were significantly reduced in the DOX group (Figures 3(h)–3(j)). Conversely, these parameters were significantly restored by treatment with Mul (Figures 3(h)–3(j)).

### 3.4. Mul Treatment-Alleviated DOX-Induced Oxidative Damage in Mice

**Figure 4:**
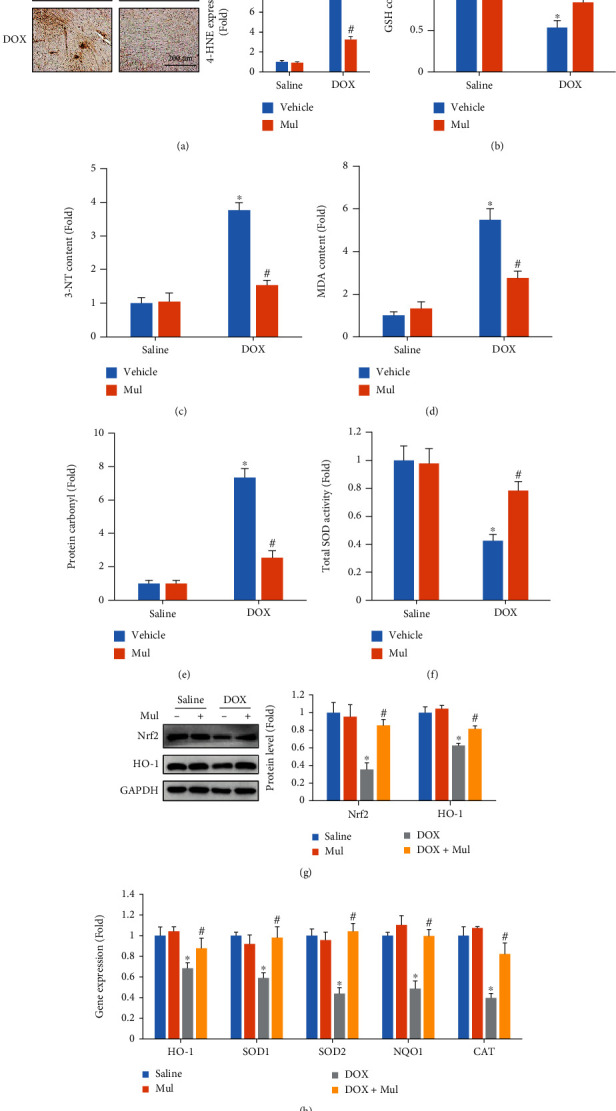
Mulberrin treatment prevented the DOX-induced oxidative damage in mice. 4-Hydroxynonenal (4-HNE) immunohistochemistry of indicated groups (a). Accumulation of the oxidative stress markers, including GSH content (b), 3-NT (c), MDA content (d), and protein carbonyl content (e), was quantified by the commercial kits. Total SOD activity (f) was also detected in DOX-treated mice. Nrf2 and HO-1 protein (g) were detected in the indicated groups. The mRNA levels of Nrf2 downstream gene products (h) were also quantified. Data are presented as mean ± SEM. *n* = 6 for each group. ^∗^*P* < 0.05 compared with the saline group; ^#^*P* < 0.05 compared with DOX alone.

As oxidative damage was a fundamental feature of acute cardiac injury induced by DOX, the effect of Mul on oxidative status was evaluated. Mul treatment also suppressed the production of myocardial lipid peroxidation in DOX-injected mice (Figure 4(a)). Upon DOX injection, GSH levels in cardiac tissues were decreased, and treatment with Mul administration almost restored the level of GSH in the hearts (Figure 4(b)). In addition, compared with the saline group, all oxidative damage markers, including 3-NT, MDA, and protein carbonyl, in heart tissues were significantly increased (Figures 4(c)–4(e)). Conversely, Mul treatment largely suppressed the elevations in these markers. We also found that the impaired SOD activity in DOX-treated hearts was improved via the treatment of Mul (Figure 4(f)). As shown in Figure 4(g), Nrf2 protein expression and the downstream HO-1 of DOX mice were assessed via western blotting, and we found that Nrf2 and HO-1 protein expressions were significantly decreased via DOX injection compared with the saline group. However, compared with the DOX group, Mul treatment significantly increased Nrf2 and HO-1 protein expression (Figure 4(g)). Further detection of the mRNA levels of Nrf2-regulated genes also suggested that Mul treatment significantly increased the mRNA expression of HO-1, SOD1, SOD2, NQO1, and CAT in DOX-treated mice (Figure 4(h)).

### 3.5. Mul Treatment-Alleviated Inflammation and Apoptosis in DOX-Treated Cardiac Tissues

**Figure 5:**
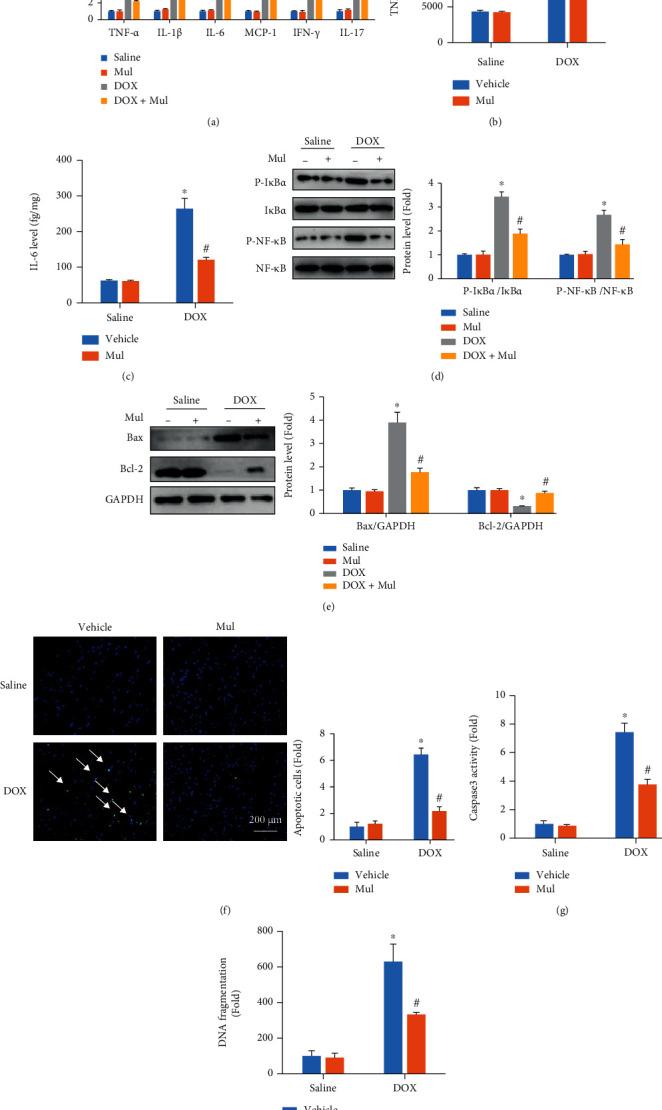
Mulberrin attenuated the expression of inflammatory cytokines in DOX-treated mice. The mRNA levels of inflammatory factors (a) were also quantified. The protein expression of TNF-*α* (b) and IL-6 (c) were detected by ELISA. The phosphorylation of NF-*κ*B and I*κ*B*α* (d) was detected in the indicated groups. The protein expression of Bax and Bcl-2 (e) was detected by western blotting. Myocardial apoptosis were evaluated by the TUNEL staining (f), caspase 3 activity (g), and DNA fragmentation test (h). Data are presented as mean ± SEM. *n* = 6 for each group. ^∗^*P* < 0.05 compared with the saline group; ^#^*P* < 0.05 compared with DOX alone.

The mRNA levels of several cytokines in heart samples were measured, and the results demonstrated that the expression of these cytokines in the hearts was significantly increased after exposure to DOX (Figure 5(a)). Treatment with Mul largely alleviated the DOX-induced inflammatory response in mice (Figure 5(a)). Further detection revealed that Mul treatment prevented the production of cardiac TNF-*α* and IL-6 in DOX-treated mice (Figures 5(b) and 5(c)). DOX induced the transactivation of NF-*κ*B, which played a key role in DOX-associated inflammation [16]. In response to DOX injection, the activated IKK complex phosphorylates I*κ*B*α*, thereby leading to nuclear accumulation of NF-*κ*B to regulate inflammatory gene expression. As indicated in Figure 5(d), the phosphorylation of I*κ*B*α* and NF-*κ*B was increased in DOX-injected hearts; these pathological alterations were not observed in Mul-treated hearts. In view of the anti-inflammatory effects of Mul, we hypothesized that Mul treatment might suppress cardiomyocyte apoptosis in mice with DOX injection. As expected, DOX increased the proapoptotic protein Bax but decreased the antiapoptotic protein Bcl-2. However, these abnormal alterations were prevented by Mul (Figure 5(e)). The number of TUNEL-positive cells was much higher in the DOX group than in the control group, and Mul largely decreased the number of these apoptotic cells (Figure 5(f)). Caspase 3 activation and DNA fragmentations in DOX hearts were increased compared with the saline group, and these effects were reduced via Mul treatment (Figures 5(g) and 5(h)).

### 3.6. Mul-Enhanced AKT Signaling Pathway in the Hearts of DOX-Treated Mice

**Figure 6:**
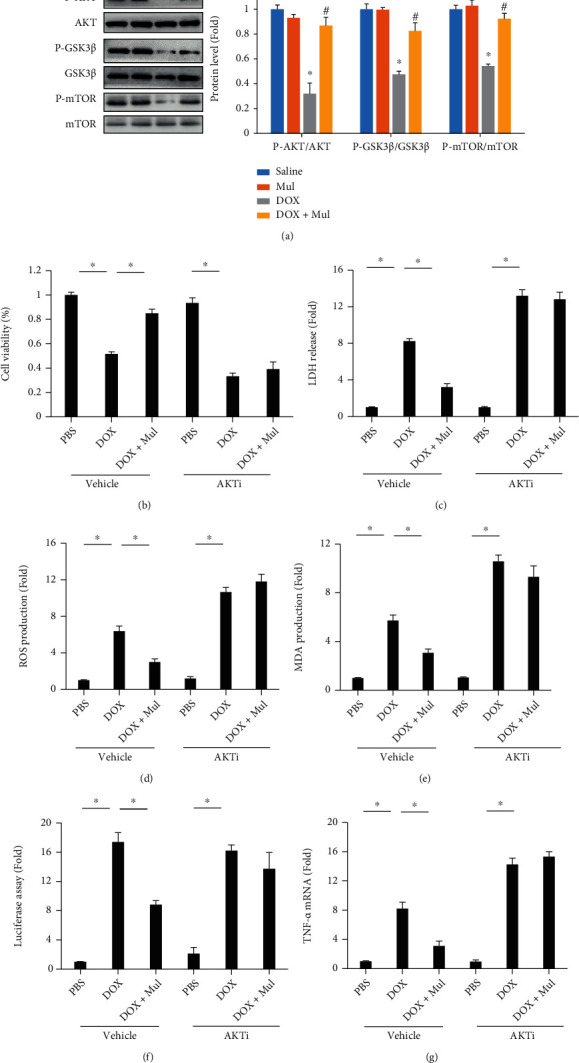
AKT inhibition abolished the anti-inflammatory and antioxidative activities of mulberrin in DOX-stimulated cells. The phosphorylation of AKT, GSK3*β*, and mTOR (a) was detected in the indicated groups. Cell viability was detected in cells treated with an AKT inhibitor (b). Cardiomyocyte injury was assayed by the release of LDH (c). The ROS level (d) and MDA content (e) were detect to reflect oxidative damage caused by DOX in cells incubated with an AKT inhibitor. Luciferase assay (f) was used to reflect NF-*κ*B transactivation in vitro. The mRNA level of TNF-*α* (g) in cells incubated with an AKT inhibitor. Data are presented as mean ± SEM. *n* = 6 for each group. For (a), ^∗^*P* < 0.05 compared with the saline group; ^#^*P* < 0.05 compared with DOX alone. For others, ^∗^*P* < 0.05 compared with the control groups.

Given that AKT was responsible for the activation of Nrf2 and that AKT activation could provide benefit against DOX-induced cardiomyocyte apoptosis [17], we asked whether Mul exerted its protective effects on DOX-induced injury through an AKT-dependent manner. DOX caused a significant decrease in the phosphorylation of AKT and downstream kinase GSK3*β* and mTOR in the heart tissues (Figure 6(a)). Mul treatment prevented the DOX-induced inactivation of the two kinases (Figure 6(a)). To test the role of AKT in the protection provided by Mul, we used a specific AKT inhibitor. As expected, AKT inhibition blocked the beneficial effect of Mul on cell viability and LDH release (Figures 6(b) and 6(c)). DOX-induced oxidative stress which was reflected by ROS and MDA production was suppressed by the treatment of Mul, and these protective effects were offset by the use of this AKT inhibitor (Figures 6(d)–6(e)). AKT inhibition blocked the beneficial effects of Mul on inflammatory response, as reflected by the luciferase assay of NF-*κ*B transactivation and TNF-*α* mRNA expression (Figures 6(f) and 6(g)).

### 3.7. AKT Signaling Was Responsible for Mul-Mediated Protective Role in the Hearts

**Figure 7:**
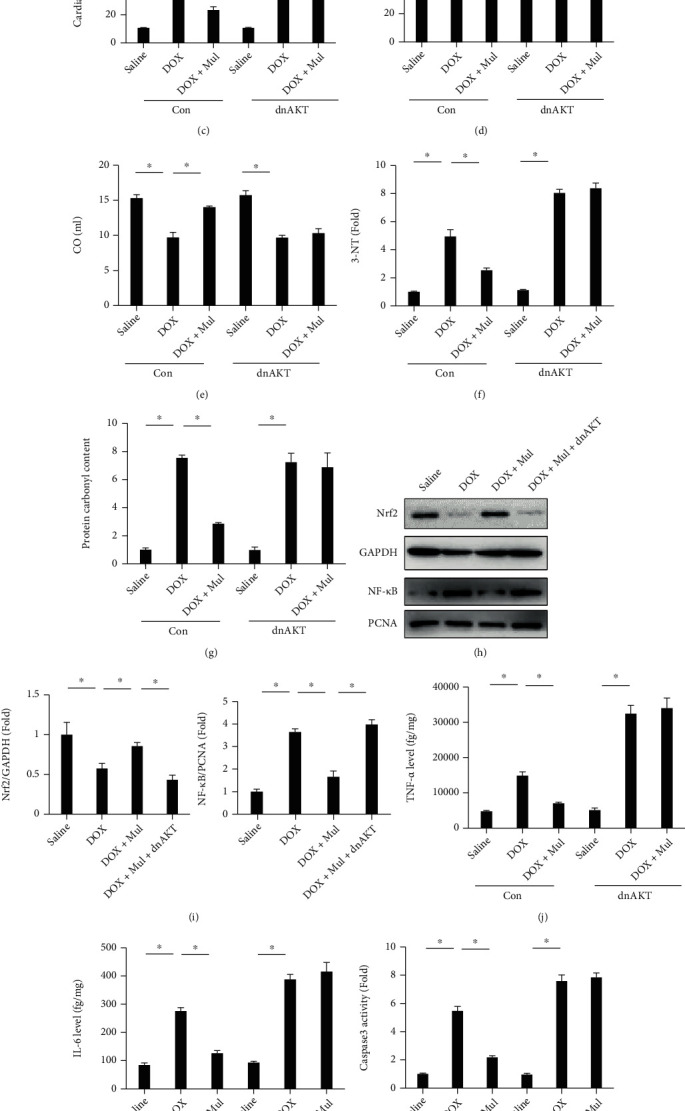
Infection of AKT-dominant negative mutant blocked the protection of mulberrin against DOX-induced cardiac injury in vivo. The phosphorylation of AKT (a) was detected via western blotting in mice at 4 weeks after AAV9-dnAKT or AAV9-Con injection. Heart weight (b) and myocardial LDH (c) were detected to evaluate DOX-induced cardiac injury. Cardiac function including ejection fraction (d) and cardiac output (e) were detected using an invasive hemodynamic monitoring. Oxidative stress markers, 3-NT (f) and protein carbonyl content (g), were quantified by the commercial kits. Nrf2 protein expression (h, i) was detected via western blotting. The nuclear accumulation of NF-*κ*B protein (h, i) was detected in an isolated nuclear fraction. The levels of inflammatory factors (j, k) were also quantified. Myocardial apoptosis was evaluated by caspase 3 activity (l) and DNA fragmentation test (m). Data are presented as mean ± SEM. *n* = 8 for each group. ^∗^*P* < 0.05 compared with the control groups.

To further verify the effect of AKT in Mul-mediated protective actions, we used an AKT-dominant negative mutant to alter the activation of AKT in the hearts. As shown in Figure 7(a), this dominant negative mutant significantly reduced myocardial AKT activation at 4 weeks after AAV9-dnAKT or AAV9-Con injection. AKT-dominant negative mutant infection almost completely abolished the Mul-mediated protective role in the hearts, as indicated by the alterations in heart weight, cardiac LDH release, and cardiac function (EF and CO) (Figures 7(b)–7(e)). DOX-induced oxidative stress, as reflected by the 3-NT content and protein carbonyl, was suppressed by the treatment of Mul. And these effects were abolished by the AKT-dominant negative mutant infection (Figures 7(f) and 7(g)). Nrf2 protein expression was decreased in response to DOX exposure but restored by the treatment of Mul. This restoration of Nrf2 was blocked by this AKT-dominant negative mutant infection (Figures 7(h) and 7(i)). Mul treatment lost its anti-inflammatory effects, as detected by the alteration of NF-*κ*B, TNF-*α*, and IL-6 levels (Figures 7(h)–7(k)). The protective effects of Mul treatment on caspase 3 activity and DNA fragmentations were also blocked by this AKT-dominant negative mutant infection (Figures 7(l) and 7(m)).

## 4. Discussion

Previous studies have confirmed a high affinity of DOX to the heart, and DOX could be accumulated in the cardiomyocytes, thus causing myocardial apoptotic loss and congestive heart failure [10]. Dexrazoxane was the only drug that was approved by FDA for the treatment of DOX-induced cardiotoxicity [18]. However, this drug had life-threatening side effects, including myelosuppression and coagulation disorder. Clearly, finding an effective approach to treat DOX-induced cardiac injury would of great clinical significance. Actually, several scavengers of ROS were capable of attenuating DOX-related cardiotoxicity in animal experiments [19, 20]. The reasons for their failure in clinical practice included low bioavailability and secondary reactions with other molecules [21]. Here, we observed that Mul treatment could attenuate DOX-induced cardiac damage and inhibit DOX-induced inflammatory response, oxidative damage, and myocardial apoptosis in mice. Supplementation of Mul might serve as a novel therapy for DOX-induced cardiotoxicity.

Emerging evidence suggested that oxidative stress and inflammatory response were the major mediators of DOX-related cardiotoxicity [22]. DOX was rapidly reduced to a semiquinone through one-electron reduction of the quinone moiety, semiquinone autoxidized, and produced superoxide anions [23, 24]. Excessive ROS production provoked oxidative damage to the lipid and protein, thus causing cardiac dysfunction [25]. In our study, multiple lines of findings suggested that DOX-induced ROS generation and oxidative damage in cardiac tissues were prevented by Mul treatment. In addition, heart samples had lower levels of endogenous antioxidant enzymes. What was more serious was that DOX significantly reduced the endogenous antioxidant, rendering the heart more vulnerable to DOX-induced oxidative stimuli. In this aspect, the restoration of antioxidant systems via the treatment of Mul might also contribute the protection of Mul against cardiotoxicity. It has been reported that Nrf2, a basic leucine zipper bZIP protein, was closely involved into DOX-induced cardiomyopathy. Nrf2 deficiency aggravated DOX-related cardiotoxicity and cardiac dysfunction in mice [26]. Conversely, activation of Nrf2 via a pharmacological agent protected against DOX toxicity through upregulation of antioxidant and antielectrophile enzyme expression in mice [27]. In our study, the expression of Nrf2 and its downstream targets was found to be decreased after DOX exposure, and treatment of Mul significantly blocked these pathological alterations, suggesting that Mul might improve antioxidant capacity in DOX-related hearts via activation of Nrf2.

Proinflammatory factors were closely involved into DOX-induced heart dysfunction [28]. Surprisingly, we demonstrated that treatment with Mul not only attenuated the DOX-induced upregulation of inflammatory factors but also inhibited the phosphorylation of I*κ*B*α* and transactivation of NF-*κ*B. However, there sounds a different voice about alteration of NF-*κ*B in DOX-induced acute cardiac injury. Riad et al. reported that DOX treatment did not induce a significant alteration of NF-*κ*B binding activity [29]. This finding could not compromise the fundamental functions of NF-*κ*B and NF-*κ*B-dependent inflammation in cardiac injury caused by DOX injection. Taken together, these data indicated that the protection of Mul against DOX-related cardiac injury might partly be attributed to the inhibitory effects on I*κ*B*α*/NF-*κ*B association and subsequent transcription of inflammatory factors.

The increase in myocardial apoptosis was another landmark of DOX-induced cardiac injury [30]. Attenuation of p53-dependent apoptosis improved cardiac function in DOX-injected mice [31]. In agreement with these findings, we observed a marked elevation in myocardial apoptosis and caspase 3 activity after DOX injection. After Mul treatment, TUNEL-positive cells, DNA fragmentation, and the Bax/Bcl-2 ratio were all decreased in DOX-injected mice. The results indicated that Mul treatment could suppress DOX-induced these apoptotic alterations in mice.

AKT inactivation played a critical role in DOX-induced cardiac injury, and restoration of AKT activity protected against cardiac apoptosis and prevented DOX-induced cardiac dysfunction [32]. Here, for the first time, we showed that Mul treatment activated the AKT signaling pathway and provided benefits against DOX-induced cardiac injury. This finding was in line with a study that reported that AKT inactivation was closely involved in the process of DOX-induced cardiotoxicity [33]. AKT inactivation resulted in GSK3*β*/FYN activation, thus promoting the nuclear exclusion of Nrf2 and its degradation in cytoplasm [34]. Here, we also reported that DOX decreased Nrf2 expression, and Mul treatment restored Nrf2 protein expression. And this restoration was blocked by the use of AKT-dominant negative mutant infection, suggesting that Mul treatment regulated Nrf2 activation through an AKT-dependent manner in DOX-treated mice.

In conclusion, these data suggested that Mul treatment attenuated DOX-induced acute cardiotoxicity via the AKT-dependent attenuation of oxidative damage, inflammation accumulation, and myocardial apoptosis. Our present findings suggest that Mul could be useful in the therapy of DOX-induced acute cardiotoxicity.

## Data Availability

The data that support the findings of this study are available from the corresponding authors upon reasonable request.
